# Eyes Absent Tyrosine Phosphatase Activity Is Not Required for *Drosophila* Development or Survival

**DOI:** 10.1371/journal.pone.0058818

**Published:** 2013-03-12

**Authors:** Meng Jin, Barbara Jusiak, Zengliang Bai, Graeme Mardon

**Affiliations:** 1 Laboratory of Developmental Immunology, School of Life Science, Shandong University, Jinan, Shandong, People’s Republic of China; 2 Program in Developmental Biology, Baylor College of Medicine, Houston, Texas, United States of America; 3 Department of Molecular and Human Genetics, Baylor College of Medicine, Houston, Texas, United States of America; 4 Department of Pathology and Immunology, Baylor College of Medicine, Houston, Texas, United States of America; 5 Department of Neuroscience, Baylor College of Medicine, Houston, Texas, United States of America; 6 Department of Ophthalmology, Baylor College of Medicine, Houston, Texas, United States of America; 7 Program in Cell and Molecular Biology, Baylor College of Medicine, Houston, Texas, United States of America; Indiana University, United States of America

## Abstract

Eyes absent (Eya) is an evolutionarily conserved transcriptional coactivator and protein phosphatase that regulates multiple developmental processes throughout the metazoans. *Drosophila eya* is necessary for survival as well as for the formation of the adult eye. Eya contains a tyrosine phosphatase domain, and mutations altering presumptive active-site residues lead to strongly reduced activities in ectopic eye induction, *in vivo* genetic rescue using the *Gal4-UAS* system, and *in vitro* phosphatase assays. However, these mutations have not been analyzed during normal development with the correct levels, timing, and patterns of endogenous *eya* expression. To investigate whether the tyrosine phosphatase activity of Eya plays a role in *Drosophila* survival or normal eye formation, we generated three *eya* genomic rescue (*eyaGR*) constructs that alter key active-site residues and tested them *in vivo.* In striking contrast to previous studies, all *eyaGR* constructs fully restore eye formation as well as viability in an *eya* null mutant background. We conclude that the tyrosine phosphatase activity of Eya is not required for normal eye development or survival in *Drosophila*. Our study suggests the need for a re-evaluation of the mechanism of Eya action and underscores the importance of studying genes in their native context.

## Introduction

*Drosophila* eye development relies on a network of retinal determination (RD) genes, which encode highly conserved transcription factors and cofactors [1]. A key member of the RD gene network is *eyes absent* (*eya*), which is necessary for survival and normal eye formation, as well as sufficient for ectopic eye induction when overexpressed [2], [3]. The four mammalian *Eya* homologues (*Eya1-4*) regulate the development of the kidneys, ears, craniofacial and skeletal structures, muscle, thymus, parathyroid, and lungs, as well as acting in the innate immune response, DNA damage repair, inhibition of apoptosis, angiogenesis, and photoperiodism [4]. Notably, *Eya1-4* are implicated in several diseases in humans, such as the multi-organ developmental disorderbranchio-oto-renal (BOR) syndrome [5], congenital cataracts [6], and late-onset deafness [7], and are overexpressed in multiple types of cancers [8]–[11].

Eya is known to act as a transcriptional coactivator as well as a protein phosphatase. The highly conserved C-terminal region of Eya, referred to as the Eya domain (ED) [12], contains tyrosine phosphatase activity of the haloacid dehalogenase family [13]–[16]. *In vitro* phosphatase assays show that Eya proteins derived from plant, mouse, and fly exhibit tyrosine phosphatase activity, although that of *Drosophila* Eya is very low and is difficult to detect [13], [14]. Multiple lines of evidence suggest that Eya tyrosine phosphatase regulates development in mammals. In humans, loss of tyrosine phosphatase activity is observed in BOR-associated mutations in *EYA1* [17], , implying that loss of tyrosine phosphatase function contributes to this disease. Over-expression of murine *Eya1*, *Eya2*, or *Eya3* results in increased proliferation, migration, invasion, and transformation of breast cancer cells. Interestingly, *Eya1/2/3* phosphatase-dead mutations attenuate induction of migration, invasion, and transformation, suggesting that the tyrosine phosphatase activity promotes tumor cell invasiveness [9]. Finally, tyrosine phosphatase activity is necessary for Eya1/2/3 to activate reporter gene expression in mammalian cell culture [15].

In *Drosophila*, cDNA-based *eya* mutant transgenes that disrupt the predicted tyrosine phosphatase active site (D493N and E728Q) have drastically decreased ability to induce ectopic eye formation and to rescue eye development in the *eya^2^* eye-specific loss-of-function mutant [13], [14]. In addition, a study based on overexpression of wild type and phosphatase-inactive *eya* transgenes in the developing eye suggests that Eya tyrosine phosphatase regulates photoreceptor axon targeting [19]. Although these findings suggest that *Drosophila* Eya tyrosine phosphatase activity may play a role during normal development, this hypothesis has not been tested using a system that accurately reproduces endogenous levels, timing, and patterns of *eya* expression.

We recently reported a study that used a genomic DNA-based rescue system to evaluate the *in vivo* significance of two predicted MAPK target sites in the Eya protein. Previous experiments using the *Gal4-UAS* system had suggested that MAPK-mediated phosphorylation activates Eya during ectopic eye development. In contrast, our genomic rescue-based study found that the two MAPK target residues of Eya are not required for normal eye development or survival in *Drosophila* [20]. In the current study, we have used the same genomic rescue strategy to gain a more accurate understanding of the role of Eya tyrosine phosphatase activity during normal *Drosophila* development. Surprisingly, we find that tyrosine phosphatase activity is not required for any known function of the Eya protein during normal *Drosophila* development.

## Materials and Methods

### Recombineering-induced Point Mutagenesis and Fly Transgenesis

We used a two-step recombineering method to create the D493N (GAT->AAT) and E728Q (GAG->CAG) point mutations in the *eya^+^GR* construct as described previously [21]. The recombineering products were sequenced and subjected to restriction enzyme fingerprint digest prior to transgenesis. We then employed φC31 to integrate constructs into *attP2* (68A) on the third chromosome [22]. Site-specific integration into *attP2* was confirmed by genomic PCR with *attP* and *attB* primers [23], and transgenic flies carrying the three different *eya* transgenes were confirmed by genomic DNA PCR sequencing. Primer sequences are available on request.

### Fly Work

Flies were raised on standard media at 25°C. Fertility of rescued flies was tested as follows: five males or females of a given genotype were mated to 10 *w^1118^* females or five males in each vial, respectively, and allowed to lay eggs for 48 hours before being discarded. Triplicate vials were set up for *eya^cli^/CyO, Df/CyO* and eya^cli^/Df; eya*GR/+. All resulting F1 progeny were counted during the first three days of eclosion.

### Histology and Immunohistochemistry

Tangential sections of three-day-old adult eyes were performed as described [24]. Images of eye sections and whole adult eyes were taken with a Zeiss AxioPlan 2 microscope and AxioVision software. Images of whole-mount adult eyes were processed with CZ Focus software.

Larval imaginal discs [25] and eye-brain complexes [26] were dissected and stained as previously described. Primary antibodies used were 1∶300 mouse anti-Eya (10H6, Developmental Studies Hybridoma Bank), 1∶400 rat anti-Elav (7E8A10, Developmental Studies Hybridoma Bank), 1∶100 mouse anti-Chaoptin (24B10, Developmental Studies Hybridoma Bank), and 1∶800 rabbit anti-β-galactosidase (ab9361, Abcam). Secondary antibodies used (1∶500 for discs, 1∶200 for brains) were Alexa Fluor 488 goat anti-mouse and anti-rat (Molecular Probes) and Cy3 goat anti-rabbit (Jackson ImmunoResearch). Photography was carried out using a Zeiss LSM 510 confocal microscope and processed with Image J software.

### Electroretinograms

Six three-day-old adults were assayed for each genotype**.** ERGs were performed as described previously [27]. Recordings were processed with AxoGraph X software.

### Statistical Analysis

For viability tests, goodness of fit was evaluated using the chi-square test. For fertility tests and scoring overshooting of axon bundles, data are presented as the mean ± standard deviation (s.d.). Statistical significance (p values) of each data set was tested using ANOVA.

## Results

### Tyrosine Phosphatase-dead Genomic Rescue Constructs

**Figure 1:**
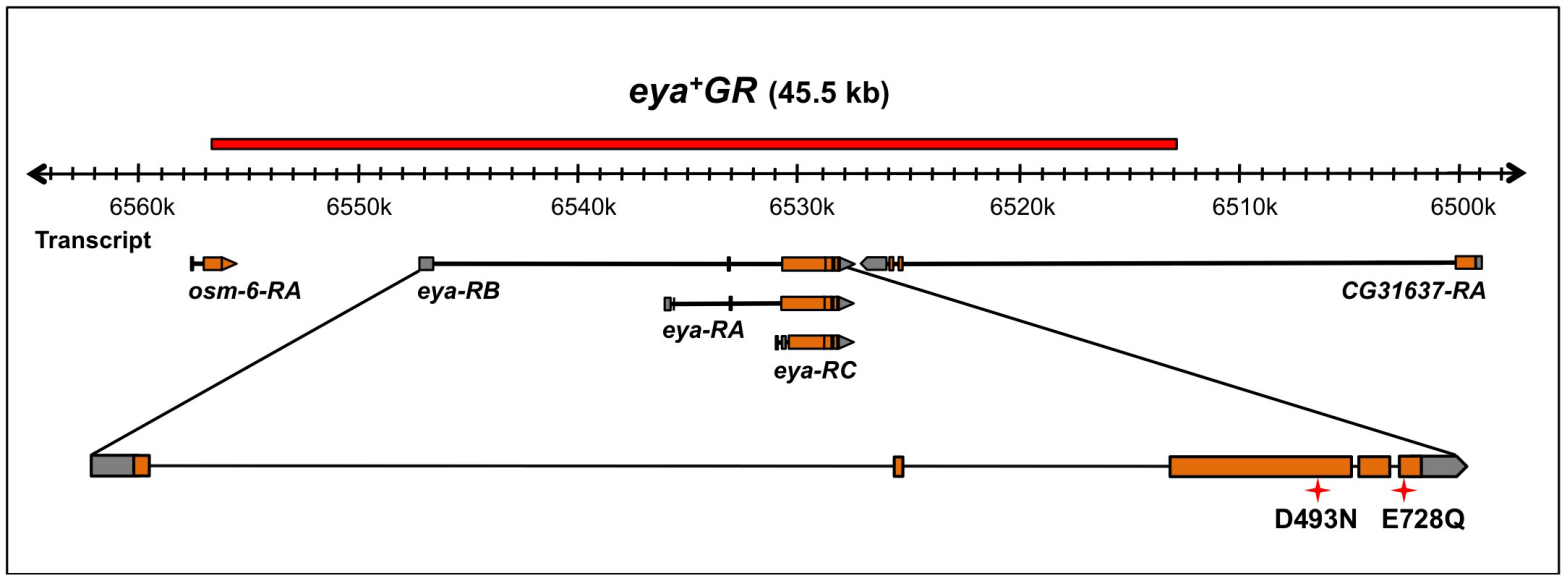
Schematic of the *eya^+^GR* transgene and *eya* locus with the position of mutations indicated. A 45.5 kb region of the genomic DNA surrounding the *eya* locus (shown as a red bar) was recombineered into *attB-P[acman]-Ap^R^*. *eya* has three alternative transcripts (*eya-RA*, *-RB*, and *-RC*). Red 4-point stars indicate the tyrosine phosphatase active site mutations; D493N and E728Q are in the 3^rd^ and 5^th^ exons of *eya*, respectively.

In this study, we utilized *eya* genomic rescue constructs (*eyaGR*, Fig. 1) and site-specific transgenesis [22], [28] to investigate whether tyrosine phosphatase activity of Eya is required for *Drosophila* eye development or survival. The wild type *eya* genomic rescue construct (*eya^+^GR*) fully rescues viability and eye formation in an *eya* mutant background, and is used as a positive control throughout our study. We generated three genomic rescue constructs encoding tyrosine phosphatase-dead Eya proteins. The residues Asp 493 and Glu 728 we chose to mutate have been reported previously to be required for Eya tyrosine phosphatase activity *in vitro* [13], [14]. Two of the point-mutant constructs, *eya^D493N^GR* and *eya^E728Q^GR*, encode a protein with a single amino-acid substitution: D493N and E728Q, respectively. In the third construct, *eya^NQ^GR*, we engineered both D493N and E728Q mutations in *eya^+^GR*. Hereafter *eya^D493N^GR*, *eya^E728Q^GR* and *eya^NQ^GR* are collectively described as eya*GR.

### eya*GR Transgenes Fully Rescue Eye Development as well as Viability in an *eya* Mutant Background

**Figure 2:**
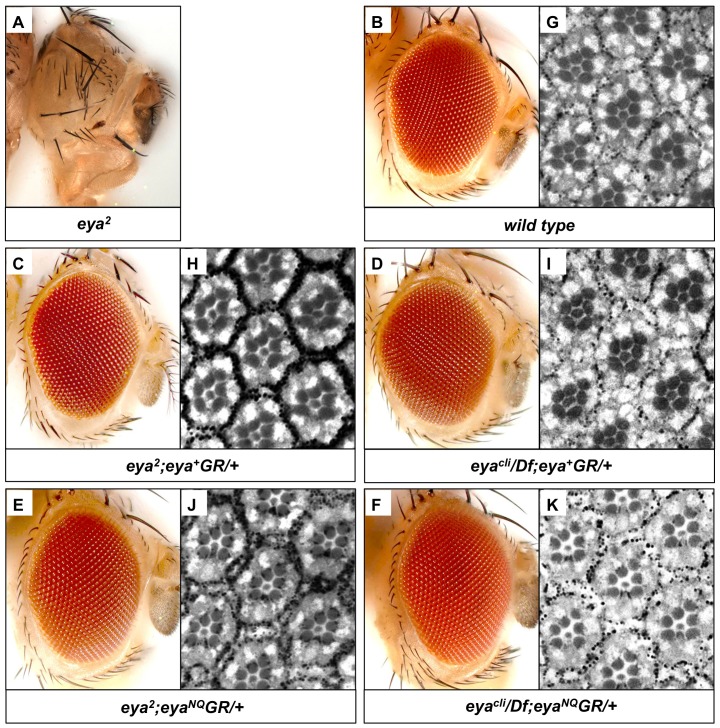
eya*GR constructs restore eye development and viability in *eya* mutants. (A) *eya^2^* homozygous adults completely lack eyes. *eya^2^* adults rescued with one copy of *eya^+^GR* (C,H) or *eya^NQ^GR* (E,J) show similar external and internal eye morphology compared with wild-type *Canton S* (B,G). A single copy of *eya^+^GR* (D,I) or *eya^NQ^GR* (F,K) can fully rescue eye morphology and survival in *eya^cli^/Df* flies, which have no endogenous *eya* function.

Previous data show that the D493N mutation in *Drosophila* Eya leads to tyrosine phosphatase inactivation, a low frequency of ectopic eye induction, and incomplete genetic rescue using the *Gal4-UAS* system [13], [14]. Likewise, murine Eya3 harboring the E478Q mutation, which is homologous to *Drosophila* E728Q, has severely decreased *in vitro* phosphatase activity [13]. Therefore, we predicted that *eya^D493N^GR* and *eya^E728Q^GR* would either rescue *eya* mutants poorly or not at all. In order to investigate the effect of mutating both catalytic residues at once, we also generated the double-mutant genomic rescue construct *eya^NQ^GR*. Hereafter we only show data for *eya^NQ^GR* since all three eya*GR constructs behaved identically. We first tested the ability of eya*GR to rescue *eya^2^* homozygous flies, which are viable and fertile but completely lack eyes due to a deletion of an enhancer required for *eya* expression during eye development [2]. We compared the external and internal eye morphology relative to that of flies rescued with *eya^+^GR* or wild type flies (Fig. 2). In contrast to our prediction, eya*GR fully rescues the eye-specific *eya^2^* mutant. Eyes of *eya^2^* adults rescued with a single copy of eya*GR reveal indistinguishable external and internal morphology from wild type.

**Figure 3:**
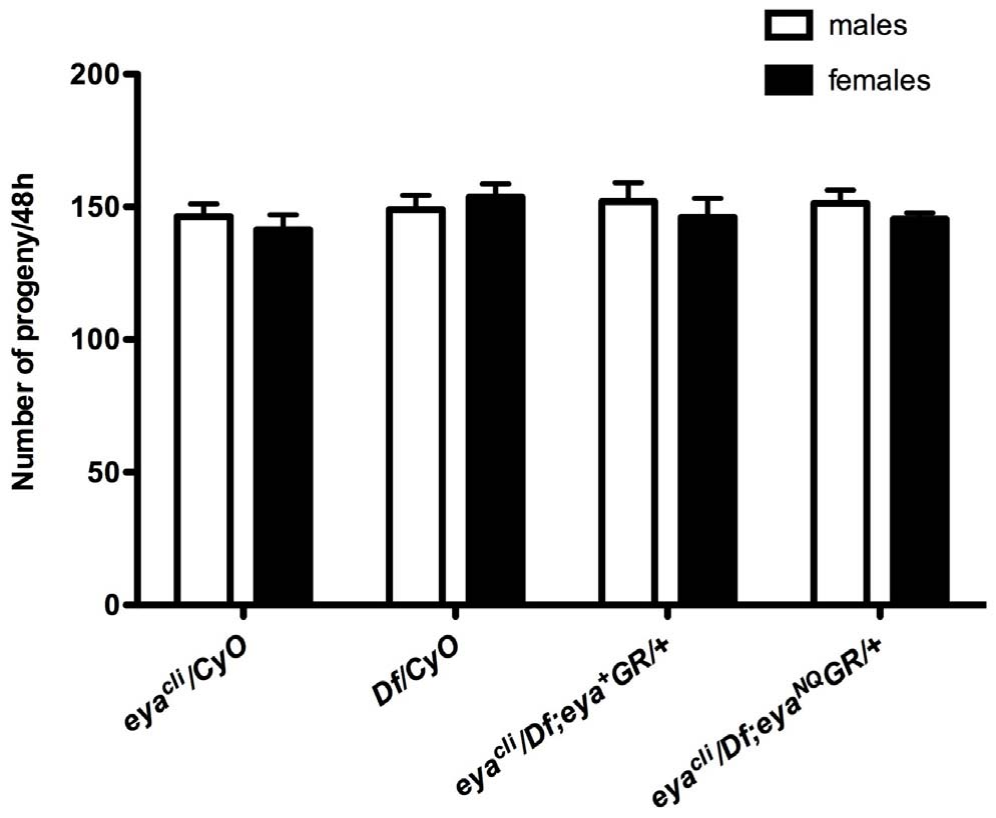
Adults rescued with eya*GR show normal fertility. Fertility assays of animals of the indicated sex and genotypes show both males and females carrying one copy of eya*GR have normal fertility. No statistically significant difference was observed compared to control *eya^cli^/CyO* and *Df/CyO* flies using ANOVA. Error bars indicate standard deviation.

To test whether eya*GR can restore viability, we performed rescue assays in a null background. The null allele *eya^clillD^* (*eya^cli^*) harbors a nonsense mutation and is embryonic lethal [29]. *Df(2L)BSC354* (hereafter referred to as *Df*) deletes the entire *eya* gene and fails to complement *eya^cli^* [30]. We crossed eya*GR into the background of *eya^cli^/Df* and found rescued adults are viable and fertile, both in males and females. Since Eya regulates muscle development [31], we also tested whether the rescued flies have normal muscle function. The eya^cli^/Df; eya*GR/+ adults appear to move and fly normally, suggesting that they have no gross defects in muscle development (data not shown). To investigate the viability of rescued flies, we crossed w; eya^cli^/CyO; eya*GR males to *w; Df/CyO* virgin females. Progeny are present at Mendelian ratios (Table 1), suggesting that the rescued flies survive as well as their heterozygous siblings. Hence, a single copy of eya*GR is functionally equivalent to a single copy of endogenous *eya* in promoting survival. Because *eya* is also required for fertility [32], we next tested whether the rescued null flies are fertile and found both males and females produce the same number of offspring as positive controls when crossed to *w^1118^* females and males (Fig. 3). Moreover, *eya* null flies carrying one copy of eya*GR have eyes indistinguishable from those of flies rescued with *eya^+^GR* or wild type, both in external and internal morphology (Fig. 2). In summary, eya*GR transgenes fully restore viability, fertility, and eye development in *eya* null mutants.

**Table 1:** Flies carrying one copy of eya*GR show normal viability.

Genotype	Non*-Cy*		*Cy*		Total	χ^2^
	Observed	Expected	Observed	Expected		
*eya^+^GR*	120	128	264	256	384	0.75
*eya^NQ^GR*	129	135	274	268	403	0.40
*eya^D493N^GR*	110	122	256	244	366	1.77
*eya^E728Q^GR*	111	114	230	227	341	0.11

Progeny from w; eya^cli^/CyO; eya*GR×*w; Df/CyO* cross are present at Mendelian ratios, indicating that rescued files carrying a single copy of eya*GR do not have a survival disadvantage compared with their heterozygous siblings. χ^2^ critical (1 d.f. p 0.05) = 3.84.

### Flies Rescued with Phosphatase-inactive Eya Show Normal Development of Larval Eye Imaginal Discs

**Figure 4:**
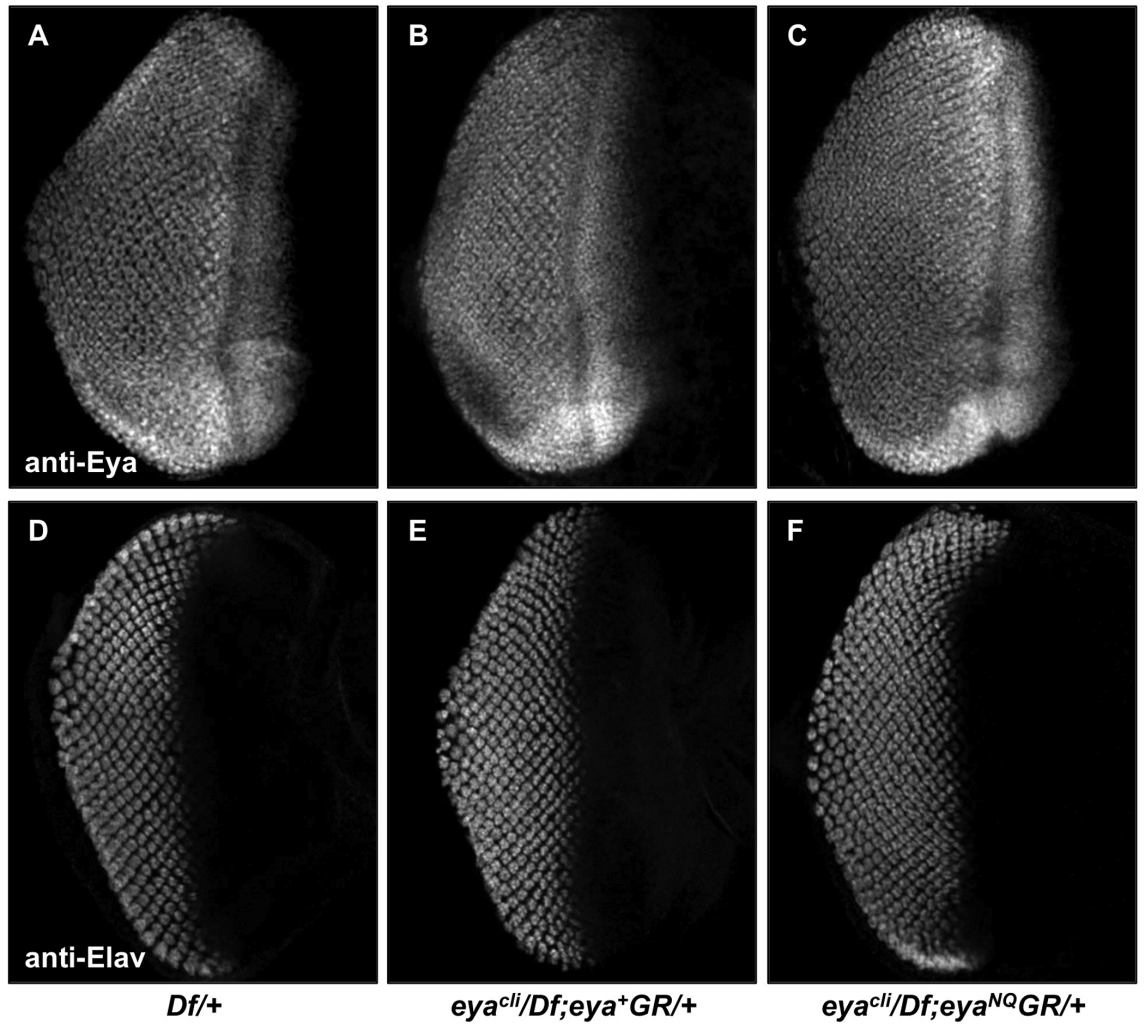
eya*GR constructs rescue larval eye imaginal disc development. Immunohistochemical detection of Eya (A–C) and the pan-neural marker Elav (D–F) in *Df/+* (A,D), *eya^cli^/Df; eya^+^GR/+* (B,E) and *eya^cli^/Df; eya^NQ^GR/+* (C,F) third instar larval eye imaginal discs. Expression patterns and levels of Eya and Elav are indistinguishable among *eya* null eye discs carrying one copy of *eya^+^GR*, *eya^NQ^GR* and *Df* heterozygotes.

The adult *Drosophila* eye arises from a larval precursor structure called the eye imaginal disc. In normal eye formation, Eya expression begins in the second instar eye disc, prior to the formation of the morphogenetic furrow (MF), which marks the onset of differentiation in the eye disc. During the third instar larval stage, the MF progresses across the eye disc,with Eya expressed in a zone of undifferentiated cells anterior to the MF as well as in progressively differentiating cells posterior to the MF. In contrast, *eya^2^* mutants have smaller eye discs due to massive apoptosis, loss of differentiation and reduced or no detectable Eya expression [2]. Given our *in vivo* rescue results, we predicted the eya^cli^/Df; eya*GR/+ larval eye discs would develop normally. As predicted, the level and pattern of Eya expression appear indistinguishable in the eye discs of positive control and rescued larvae (Fig. 4). In addition, the eye discs of rescued flies are normal in size. We also visualized Elav, a neuron-specific antigen [33], to investigate photoreceptor differentiation. Third instar eye imaginal discs from *Df* heterozygotes and rescued flies all yield similar Elav expression patterns (Fig. 4), implying that the tyrosine phosphatase-inactive mutations do not adversely affect differentiation of photoreceptors. To summarize, one copy of eya*GR is sufficient to support normal larval eye disc development.

### eya*GR Rescued Flies have a Normal Visual Response

**Figure 5:**
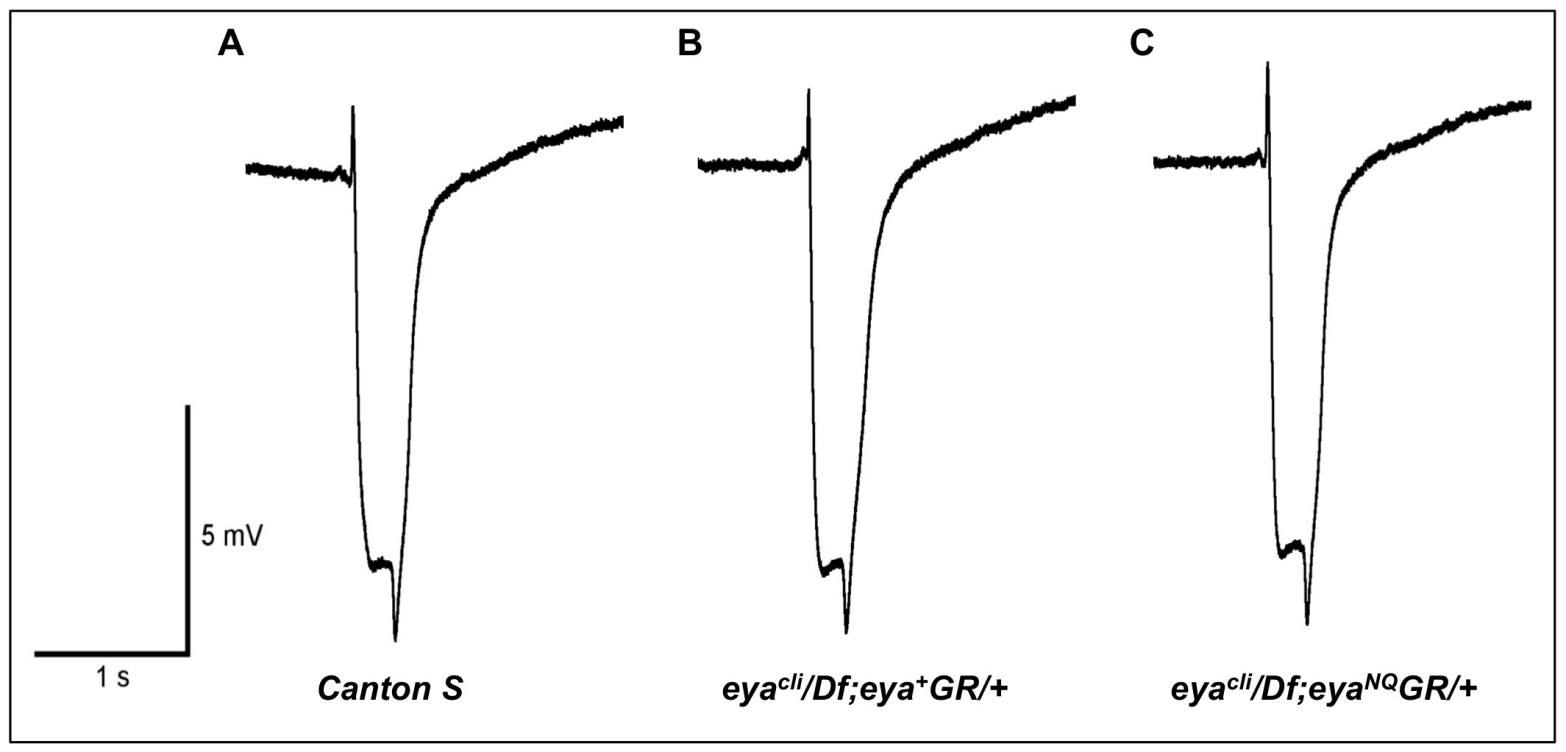
Mutations in the tyrosine phosphatase active site of Eya do not affect response to light. ERG recordings from wild-type *Canton S* (A), *eya^cli^/Df; eya^+^GR/+* (B) and *eya^cli^/Df; eya^NQ^GR/+* (C) 3-day-old retinas show similar curves and suggest rescued animals have a normal light response.

The results described above suggest that the tyrosine phosphatase catalytic residues Asp 493 and Glu 728 are not required for normal eye formation. To test whether these mutations affect eye function in rescued adults, we performed electroretinograms (ERG), a physiological assay of retinal function. Three-day-old wild type, *eya^cli^/Df; eya^+^GR/+* and eya^cli^/Df; eya*GR/+ files were assayed, and all showed indistinguishable ERG recordings (Fig. 5), indicating that Eya tyrosine phosphatase activity is dispensable for adult eye function.

### Photoreceptor Axon Projections are Normal in Adults Rescued with eya*GR

**Figure 6:**
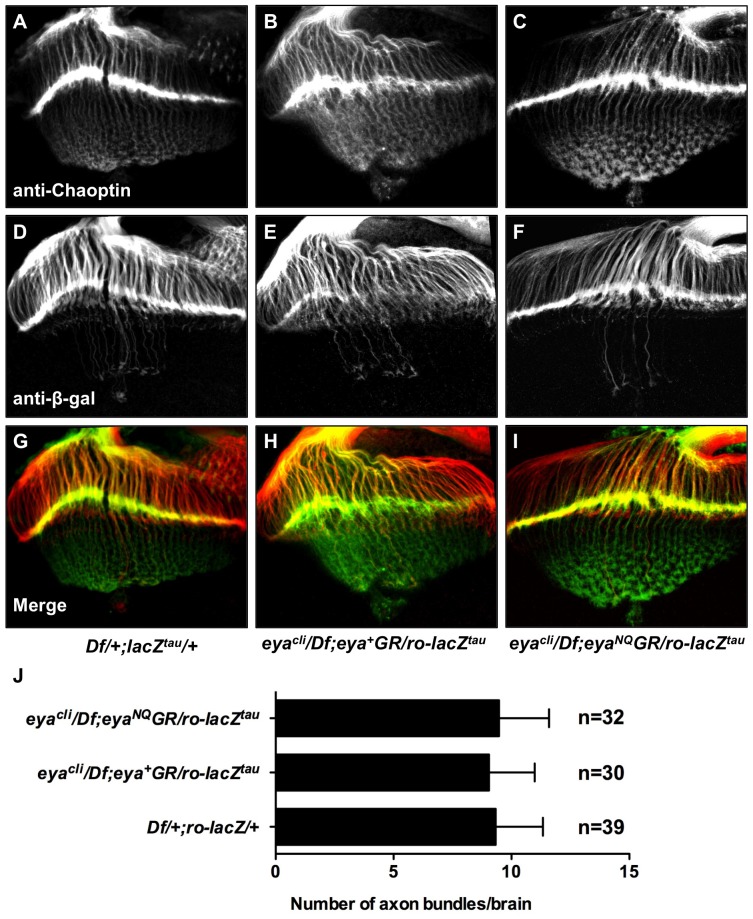
Animals rescued with eya*GR have normal photoreceptor axon projections. Third instar eye-brain complexes from *Df/+; ro-lacZ^tau^/+* (A,D,G), *eya^cli^/Df; eya^+^GR/ro-lacZ^tau^* (B,E,H), and *eya^cli^/Df; eya^NQ^GR/ro-lacZ^tau^* (C,F,I) stained with anti-Chaoptin and anti-β-galactosidase. (A–C) Projections from photoreceptors R1–8 are visualized by using mAb24B10 (anti-Chaoptin). (D–F) Projections from photoreceptors R2–R5, which terminate in the lamina, are revealed using *ro-lacZ^tau^* (anti-β-galactosidase). (G-I) Merge of channels. *eya^NQ^GR* rescued animals show normal photoreceptor axon projections, similar to flies rescued with *eya^+^GR* and *Df/+; ro-lacZ^tau^/+*. (J) 30–40 eye-brain complexes were scored for overshooting axon bundles by investigators who were blind to the genotype. The average number of overshooting axon bundles per brain is the same for *Df/+; ro-lacZ^tau^/+*, *eya^cli^/Df; eya^+^GR/ro-lacZ^tau^* and eya^cli^/Df; eya*GR/ro-lacZ^tau^. n, number of brains scored. Error bars indicate standard deviation.

Finally, previous studies have also shown that protein tyrosine phosphatase activity contributes to the regulation of photoreceptor axon targeting [19], [34]. We therefore asked if Eya phosphatase function mediated by D493 and E728 is required for normal axon targeting. We used the reporter line *ro-lacZ^tau^*, which is driven by the R2–R5 specific *rough* enhancer and encodes a Tau-β-galactosidase fusion protein that marks R2–R5 photoreceptor axons [34]. Immunohistochemistry shows eya*GR rescued animals have normal photoreceptor axon projections (Fig. 6). Moreover, the average number of overshooting axon bundles per brain is the same for each genotype (Fig. 6). Based on these axon projection and the ERG results, we conclude that eye function is not affected by loss of Eya tyrosine phosphatase activity.

## Discussion

As a dual-function protein, Eya has been best characterized as a transcriptional coactivator [35]–[37]. The second function – phosphatase activity – needs further investigation. In this study, we show that mutations in the tyrosine phosphatase active site of Eya do not affect *Drosophila* survival or normal eye development. However, previous studies suggest that the Eya domain (ED), in which the tyrosine phosphatase active site is located, is required for other functions during development. First, lethal missense and nonsense mutations within the ED of *Drosophila* Eya have been reported [38], indicating that this domain is indeed required for normal Eya function. Second, cell culture and GST pull-down studies have shown that the *Drosophila* ED binds to the transcription factor Sine oculis (So/Six), perhaps serving to recruit Eya to target genes, where it can act as a transcriptional co-activator within an Eya/So complex [18], [38]. Similarly, the ED of mammalian EYA can also bind to the SIX proteins [35], [39]. Taken together, these results suggest that while the tyrosine phosphatase activity of the ED is no longer required in *Drosophila*, it appears that some other function of the ED has either been acquired or retained since the divergence of insects and vertebrates.

cDNA-based studies have also suggested that an internal proline-serine-threonine-rich (PST) domain in Eya is required for transcriptional activation in cell culture reporter assays and for efficient induction of ectopic eyes *in vivo* [36]. Consistent with these results, our genomic rescue assays also show that the PST domain is required for *Drosophila* survival as well as normal eye development (data not shown; to be reported elsewhere). These results validate our genomic rescue system and indicate that, unlike the transactivation function, Eya tyrosine phosphatase activity is not necessary for *Drosophila* normal development.

The differences between our findings based on a genomic rescue strategy and previous assays using ectopic eye induction and cDNA-based *Gal4-UAS* genetic rescue assays may be observed for several reasons. First, normal eye formation and reprogramming of non-retinal tissue into an ectopic eye involve overlapping but distinct genetic programs, as suggested by previous studies [40]. Second, the *Gal4-UAS* system does not recapitulate the wild-type pattern or timing of *eya* expression while our genomic rescue constructs are likely to contain the full complement of regulatory sequences needed, allowing the transgene to be expressed in the same spatiotemporal pattern as the endogenous gene of interest. Evidence supporting the genomic rescue strategy comes from our findings that, unlike ectopic eye formation in *Drosophila*, normal eye development and survival are unaffected by mutations in two previously studied MAPK target residues of Eya using genomic rescue constructs [20]. Third, in contrast to the random integration of the P-element mediated *UAS-eya* transgenes used in previous studies, we used site-specific integration, minimizing genomic position effects on different constructs. Previous studies have reported that the extent of rescue is indeed highly variable among four independent insertion lines of a *UAS-eya^D493N^* transgene [14]. Finally, although recent studies have shown that an *eya^D493N^* transgene fails to rescue *eya^2^* using site-specific heat shock-mediated induction [41], this approach has several drawbacks, including inaccurate timing, patterns, and levels of transgene induction [42]. In other words, no heat shock regimen is likely to drive transgene expression timing, patterns, or levels in a manner approaching that of the endogenous *eya* gene. This report highlights the importance of the genomic rescue system for evaluating the function of specific protein domains, motifs, and even single residues or base pairs *in vivo*.

Although our studies suggest that Eya tyrosine phosphatase is dispensable for *Drosophila* development under standard laboratory conditions, it remains possible that Eya tyrosine phosphatase contributes to the animal’s response to environmental stressors. Previous studies reported that murine Eya1 and Eya3 can dephosphorylate H2AX, a histone variant associated with DNA damage, promoting efficient DNA repair [43]. Since *Drosophila* has an H2AX homologue, it will be interesting to determine if *Drosophila* Eya is also able to dephosphorylate H2AX. If this is the case, then our *eya* null mutants rescued with phosphatase-inactive eya*GR may be more sensitive to conditions that cause DNA damage.

To summarize, contrary to current models, the tyrosine phosphatase activity of Eya is dispensable for all known Eya functions in *Drosophila*. Whether the tyrosine phosphatase activity is required for mammalian Eya function *in vivo* remains to be tested. Our studies shed new light on a protein that plays a key role in a variety of developmental processes throughout the metazoans.
